# Prolonged Exposure to a Mer Ligand in Leukemia: Gas6 Favors Expression of a Partial Mer Glycoform and Reveals a Novel Role for Mer in the Nucleus

**DOI:** 10.1371/journal.pone.0031635

**Published:** 2012-02-20

**Authors:** Justine Migdall-Wilson, Christine Bates, Jennifer Schlegel, Luis Brandão, Rachel M. A. Linger, Deborah DeRyckere, Douglas K. Graham

**Affiliations:** Department of Pediatrics, University of Colorado Anschutz Medical Campus, Aurora, Colorado, United States of America; Florida International University, United States of America

## Abstract

Mer tyrosine kinase is ectopically expressed in acute lymphoblastic leukemia and associated with enhanced chemoresistance and disease progression. While such effects are generally ascribed to increased engagement of oncogenic pathways downstream of Mer stimulation by its ligand, Gas6, Mer has not been characterized beyond the scope of its signaling activity. The present study explores Mer behavior following prolonged exposure to Gas6, a context similar to the Gas6-enriched microenvironment of the bone marrow, where a steady supply of ligand facilitates continuous engagement of Mer and likely sustains the presence of leukemic cells. Long-term Gas6 exposure induced production of a partially N-glycosylated form of Mer from newly synthesized stores of protein. Preferential expression of the partial Mer glycoform was associated with diminished levels of Mer on the cell surface and altered Mer localization within the nuclear-soluble and chromatin-bound fractions. The presence of Mer in the nucleus is a novel finding for this receptor, and the glycoform-specific preferences observed in each nuclear compartment suggest that glycosylation may influence Mer function within particular subcellular locales. Previous studies have established Mer as an attractive cancer biologic target, and understanding the complexity of its activity has important implications for potential strategies of Mer inhibition in leukemia therapy. Our results identify several novel features of Mer that expand the breadth of its functions and impact the development of therapeutic modalities designed to target Mer.

## Introduction

The Mer receptor tyrosine kinase (RTK)—also known as MerTK, Nyk, and Tyro12—mediates a spectrum of physiological functions, including platelet aggregation, macrophage clearance of apoptotic cells, cytokine release, and cell proliferation and survival [1]. Many of the intracellular signaling events that influence these functions occur downstream of Mer activation upon engagement with its ligand, Gas6. This vitamin K-dependent molecule also serves as the common ligand for Axl and Tyro3 [2], two other transmembrane receptors sharing homology with Mer in the extracellular regions and a conserved sequence within the tyrosine kinase domain. Collectively, these three proteins compose the TAM subfamily of RTKs [1].

While their normal expression is critical in maintaining cell function, aberrant levels of TAM receptors and their ligands have been reported in numerous cancers and are often associated with poor prognostic indicators [1], [3], [4]. Mer is ectopically expressed in acute lymphoblastic leukemia (ALL), the most common pediatric malignancy, both within subsets of B- and T-ALL [5], [6]. Mer promotes oncogenesis in lymphocytes—which normally do not express Mer [5], [7]—and confers resistance to chemotherapy-induced apoptosis in leukemia and other cancer types [8], [9]. Furthermore, shRNA-mediated Mer inhibition delays disease onset and improves drug response in a murine xenograft model of leukemia [10].

The oncogenic effects associated with Mer are largely attributed to the increased activation of pro-survival and proliferative pathways observed in response to Mer stimulation, including those driven by MAPK/Erk and PI3K/Akt [1], [2], [8], [11]. Engagement of downstream signaling pathways, which occurs transiently *in vitro*, is also believed to persist *in vivo* due to the presence of Gas6 in the plasma [12]–[16] and bone marrow [17]–[19]. However, such signaling events—currently regarded as the primary mechanisms underlying the oncogenicity of Mer—have only been defined by short-term (i.e. 10–60 minutes) stimulation of Mer. Beyond these signaling-focused studies, much remains unknown about receptor behavior and the mechanisms influencing functional consequences associated with aberrant Mer expression. We thus used an *in vitro* model of prolonged Gas6 exposure to study Mer within a more physiologically relevant context similar to the perpetually Gas6-replete environment described to exist in both pathophysiologic and normal conditions.

Our initial investigations revealed that long-term Gas6 exposure induced preferential expression of a partial Mer glycoform normally existing at minor levels relative to the fully glycosylated receptor. Despite its partially N-glycosylated nature, the Gas6-favored Mer glycoform displayed several features indicating that it was not merely an ineffectual precursor to the fully glycosylated protein. In the process of elucidating the mechanisms underlying receptor modification, we identified a relationship between Mer glycosylation and its subcellular localization, which led to an unexpected observation of Mer expression within the nuclear compartments. This is the first report to demonstrate localization of Mer—or any of the TAM receptors—in the nucleus. Not only does this novel finding expand our understanding of Mer as a cell surface receptor to that of a potential gene expression regulator, but it also broadens the realm of available methods of inhibition in the ongoing search for targeted therapies against leukemia.

## Results

### Prolonged Gas6 exposure induces a smaller molecular weight form of Mer

**Figure 1:**
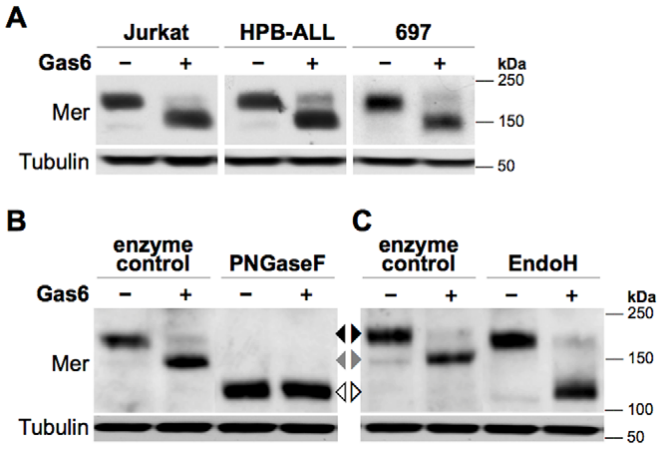
Long-term exposure to Gas6 induces expression of a partially *N*-glycosylated form of Mer. Whole-cell lysates were collected from human leukemia cell lines cultured in the presence of 200 nM Gas6 (+) or vehicle control (−) for 18 hours and Mer expression was determined by western blot. Blots were also probed with an anti-Tubulin antibody to confirm similar loading. (**A**) In human ALL cell lines, Gas6-treated cells express a major form of Mer with a faster electrophoretic mobility (∼150 kDa) than Mer in control-treated cells (∼180 kDa). (**B**) Jurkat cell lysates were digested with PNGaseF or incubated under the same conditions without PNGaseF (enzyme control) prior to SDS-PAGE. (**C**) Jurkat cell lysates were digested with EndoH or enzyme control prior to SDS-PAGE. The non-glycosylated (□), partially glycosylated (□), and fully glycosylated (□) Mer glycoforms are indicated between panels B and C.

Three human ALL cell lines—697 (B-ALL), Jurkat (T-ALL), and HPB-ALL (T-ALL)—were used in our investigations. Cells were cultured in the presence of 200 nM Gas6, a concentration sufficient for Mer activation [8], [20], and collected after 18 hours. Total protein from whole-cell lysates was separated by SDS-PAGE and Mer was detected by western blotting with an antibody specific to its extracellular domain (Figure 1A). While the majority of Mer from control samples existed as a 180-kDa band, Gas6-treated cells predominantly expressed a form of Mer with a faster electrophoretic mobility and approximate molecular weight of 150 kDa. All three ALL lines expressed this smaller molecular weight form of Mer following prolonged Gas6 exposure, indicating that this was not a cell line-specific effect.

### Gas6 favors expression of a partial Mer glycoform

Mer contains 14 putative N-linked glycosylation sites in its extracellular domain [21], [22]. Based on this and previous reports of glycosylation-related differences in Mer size [5], [23], we sought to determine if this same modification accounted for the ligand-responsive decrease in molecular weight. Indeed, removal of all N-glycosylation abolished the shift in Mer mobility between control- and Gas6-treated cells (Figure 1B): PNGaseF digestion of Jurkat lysates reduced Mer from both samples to approximately 110 kDa, the approximate predicted molecular weight of the non-glycosylated protein [21].

Treatment of lysates with EndoH, an endoglycosidase that removes only mannosylated glycans [24], reduced the Gas6-responsive Mer glycoform (150 kDa) to a similar size as that seen with PNGaseF (Figure 1C). In addition, the partial Mer glycoform expressed as a minor form in control cells (150 kDa, *first lane*) was also susceptible to EndoH, indicating that the N-glycans on both partial glycoforms terminated in high mannose. In contrast, the largest Mer glycoform (180 kDa) predominating in control cells displayed minimal sensitivity to EndoH, a characteristic commonly observed in proteins containing more complex N-glycan modifications [25].

### Expression of the partial Mer glycoform occurs in a time- and Gas6-dependent manner

**Figure 2:**
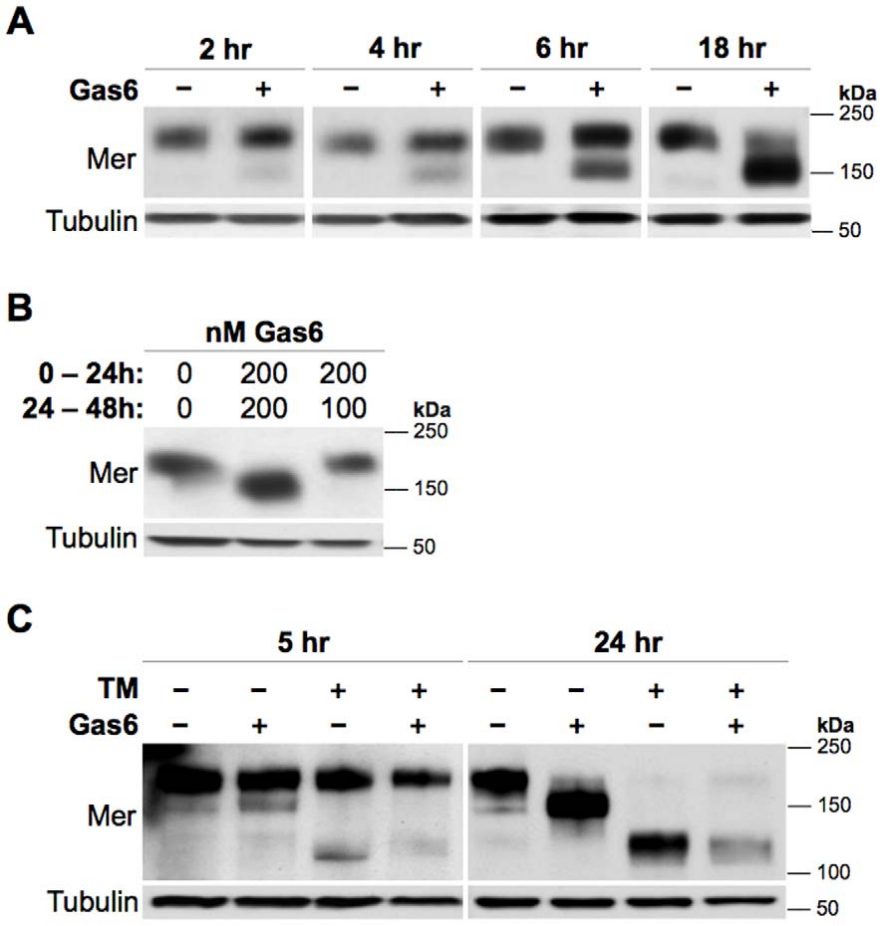
Gas6 induces expression of the partial Mer glycoform in a time- and concentration-dependent manner from a newly synthesized protein. Mer expression was detected by western blot of whole-cell lysates prepared from Jurkat cells. Blots were also probed with an anti-Tubulin antibody to assess loading. (**A**) Equal densities of cells were treated with vehicle control (−) or 200 nM Gas6 (+) and lysed after the indicated exposure times. (**B**) 24 hours after inducing expression of the partial Mer glycoform with 200 nM Gas6, cells were divided into two wells and cultured in media containing 200 or 100 nM Gas6 for an additional 24 hours prior to lysis. Control-treated cells were cultured under the same conditions. The Gas6 concentration during each of the two 24-hour intervals is indicated above the corresponding lane. (**C**) Cells were treated with 1 µg/ml tunicamycin (TM, +, *top*) or 0.1% DMSO vehicle control (−, *top*) for 1 hour prior to addition of 200 nM Gas6 (+, *bottom*) or vehicle control (−, *bottom*). Cells were lysed 5 or 24 hours after Gas6 exposure.

We next aimed to characterize the processes underlying Gas6-favored expression of the partial Mer glycoform. Western blot analysis revealed that enhanced expression of the partial glycoform developed within 2 hours of Gas6 exposure and progressed into the major form by 18 hours (Figure 2A). 200 nM Gas6 was sufficient to produce this change in glycoform preference under both serum-deprived and -complete conditions (Figure S1A), and a single dose of Gas6 sustained expression of the partial glycoform for at least 96 hours (Figure S1B). However, after inducing partial glycoform expression with a 24-hour exposure to 200 nM Gas6, subsequent reduction of Gas6 concentration to 100 nM restored expression of the full glycoform within 24 hours (Figure 2B).

These findings indicated that selective replacement of fully glycosylated Mer with the partial glycoform, likely a consequence of continued receptor engagement, required a minimum Gas6 concentration. The dose-dependent nature of this behavior is consistent with a requirement for kinase activation, as 100 nM Gas6 is not sufficient to activate Mer *in vitro*. However, prolonged exposure to 200 nM Gas6 still favored production of the partial glycoform in cells expressing a kinase-dead form of Mer (Figure S2). Additionally, we found that H_2_O_2_—which also activates Mer in a similar, dose-dependent fashion as Gas6 [26]—did not alter glycoform expression preference upon long-term exposure (data not shown), further suggesting that this process is mediated by kinase-independent mechanisms.

### The Gas6-favored Mer glycoform is produced from newly synthesized protein

To further elucidate the molecular basis of this process, we sought to determine whether the Gas6-responsive Mer glycoform resulted from a preexisting, partially deglycosylated protein or from *de novo* partial glycosylation of a newly synthesized protein. Cells were treated with tunicamycin (TM)—a naturally occurring antibiotic that inhibits core glycan synthesis and thus completely blocks glycosylation of all newly translated proteins [27]—prior to adding Gas6. If the Gas6-responsive glycoform resulted from partial deglycosylation of a pre-existing protein, TM would have no effect; conversely, if the partial Mer glycoform arose from glycosylation of a newly synthesized protein, TM would block this process and prevent its favored production in the presence of Gas6. In both Gas6- and control-treated cells, TM prevented formation of the partial glycoform (Figure 2C), a feature observed within 5 hours of Gas6 exposure. After 24 hours, all TM-treated cells predominantly expressed the 110-kDa, non-glycosylated form of Mer. These results demonstrate that addition of core glycans to newly synthesized proteins—rather than partial deglycosylation of preexisting proteins—was the critical mechanism required for formation of the partial glycoform.

### Predominant expression of the partial Mer glycoform is associated with altered downstream signaling

**Figure 3:**
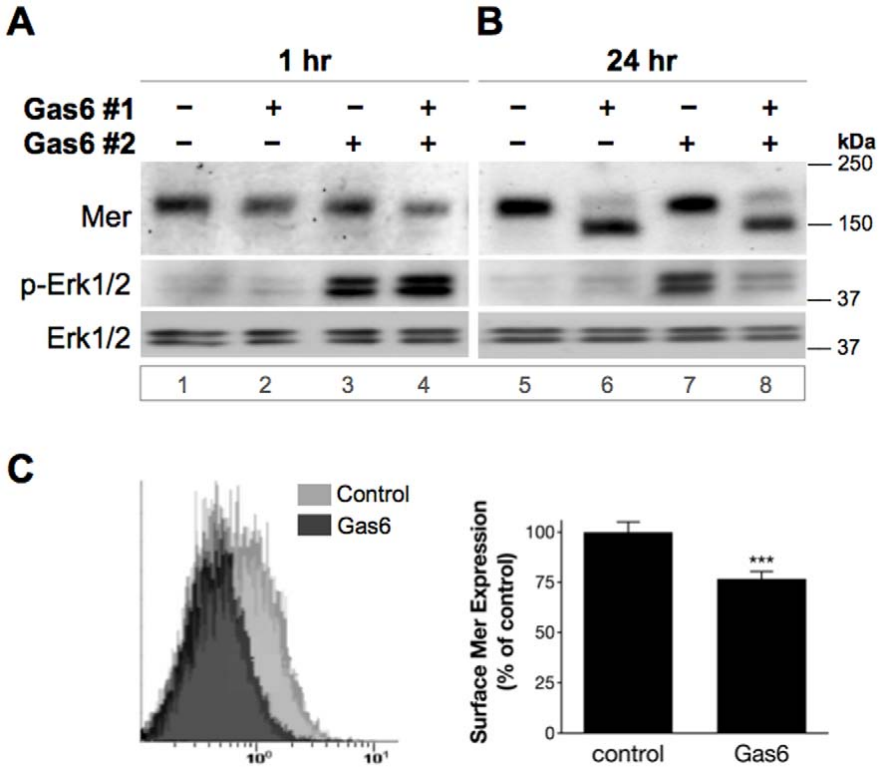
The Gas6-favored Mer glycoform is associated with altered downstream signaling and reduced surface expression. Jurkat cells were initially treated with 200 nM Gas6 (+) or control (−) for 1 hour (**A**) or 24 hours (**B**). After this first exposure (*Gas6 #1*), cells received a second dose of the same treatments, spiked in for 10 minutes (*Gas6 #2*), and were then lysed to evaluate expression of the indicated proteins by western blot (“p-” represents a phosphorylated protein). Lane numbers are designated below blot images. Cells treated with vehicle only at the time of the second exposure (*lanes 1, 2* and *5, 6*) displayed equally low levels of p-Erk1/2, indicating that basal levels of Erk activity had been restored since the time of initial Gas6 stimulation. (**C**) Surface expression of Mer was assessed by flow cytometry of Jurkat cells exposed to 200 nM Gas6 or vehicle control for 24 hours. *Left:* Representative histogram of Mer expression as a function of PE intensity (*x*-axis). *Right:* MFI (median fluorescence intensity) values expressed as percent surface expression in Gas6-treated relative to control samples. Mean values and standard deviations (SD) derived from 5 independent experiments are shown (***p<0.0001, two-tailed unpaired t-test, 99% CI).

Previous *in vitro* studies have shown that Mer stimulation results in increased Erk phosphorylation, with peak activation occurring within 10 minutes of Gas6 exposure [2], [11], thus establishing it as a useful downstream readout of Mer activation. To investigate how favored expression of the partial Mer glycoform affected downstream signaling, Erk phosphorylation (p-Erk1/2) was evaluated in cells predominantly expressing the full (Figure 3A) versus partial (Figure 3B) Mer glycoform. After an initial exposure to 200 nM Gas6 or control for either 1 or 24 hours (*Gas6 #1*), Jurkat cells were exposed to a second dose of the same treatments directly spiked in for 10 minutes (*Gas6 #2*), and then lysed for western blot analysis. Following the initial 1-hour exposure to vehicle control or 200 nM Gas6, cells treated with vehicle only during the second exposure displayed low levels of p-Erk1/2 (*lanes 1 and 2*), indicating that the ligand-responsive increase in Erk phosphorylation—observed 10 minutes after Gas6 treatment (*lanes 3 and 4*) and consistent with previous reports—occurred transiently, and Erk activity was restored to basal levels within an hour of initial Gas6 stimulation. When the majority of Mer still existed as the full glycoform 1 hour after the first exposure, Gas6 stimulation during the second exposure time elicited a robust Erk response in doubly stimulated cells (*lane 4*, which contained a total of 400 nM Gas6 for the second exposure) similar to that observed in singly stimulated cells that had first been exposed to vehicle control (*lane 3*, which contained 200 nM Gas6 for the second exposure). In contrast, when the majority of Mer existed as the partial glycoform 24 hours after initial Gas6 exposure, Erk phosphorylation was diminished following a second Gas6 stimulation (*lane 8*) relative to the increased Erk activity observed in cells stimulated for 10 minutes with Gas6 after first being exposed to vehicle control for 24 hours (*lane 7*). These data indicate that predominant expression of the partial Mer glycoform, rather than saturation with Gas6, correlates with the altered signaling patterns observed downstream of Mer activation.

### Reduced Mer surface expression results from partial glycosylation

Among several factors potentially influencing this reduction in Erk activation was the possibility that less Mer was available for Gas6 engagement at the cell surface. To address this, surface expression of Mer was measured by flow cytometry after exposing cells to 200 nM Gas6 or vehicle control for 24 hours (Figure 3C). Surface levels of Mer were decreased by approximately 25% in Gas6-treated cells relative to control, suggesting that the altered signaling observed in the presence of the partial Mer glycoform may, at least in part, result from its reduced surface expression.

**Figure 4:**
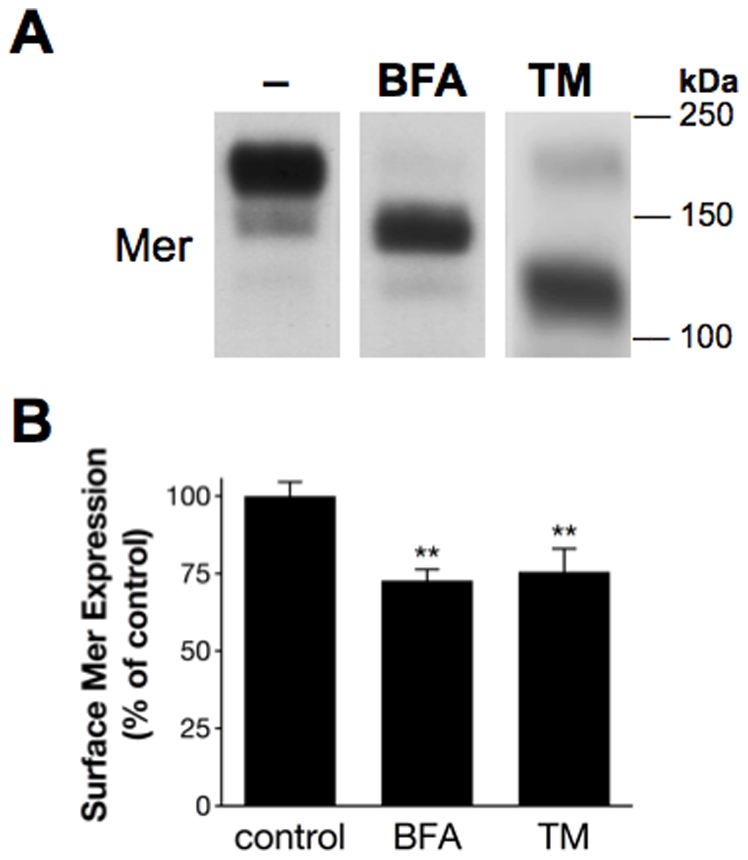
Reduced surface expression of Mer results from incomplete glycosylation. Jurkat cells were cultured in the presence of 5 µg/ml Brefeldin A (BFA), 1 µg/ml TM, or 0.1% DMSO vehicle control (−) for 24 hours. (**A**) Western blot detection of Mer from whole-cell lysates. (**B**) Surface expression of Mer was measured by flow cytometry, and MFIs from BFA- and TM-treated samples were normalized to control MFI for each experiment. Mean values and SD derived from 3 independent experiments are shown (**p<0.005, 1-way repeated measures ANOVA followed by Bonferroni post-tests).

As previous studies have demonstrated that incomplete glycosylation can impair delivery to the cell surface [28], we next explored whether partial glycosylation alone was sufficient to limit Mer expression on the cell surface. Treatment with Brefeldin A (BFA), which disrupts trafficking from the endoplasmic reticulum (ER) to the Golgi and thus prevents glycosylation beyond ER-localized mannosylation [29], resulted in a partially glycosylated form of Mer with a molecular weight similar to the minor glycoform expressed in control cells (Figure 4A). BFA-restricted partial glycosylation, as well as complete glycosylation inhibition by tunicamycin (TM), both resulted in a similar approximate 25% decrease in surface Mer expression observed by flow cytometry (Figure 4B). These data suggest that reduced expression of surface Mer was a primary effect of incomplete glycosylation and a secondary consequence of prolonged Gas6 exposure.

### Continuous Gas6 exposure alters Mer expression in the nucleus

**Figure 5:**
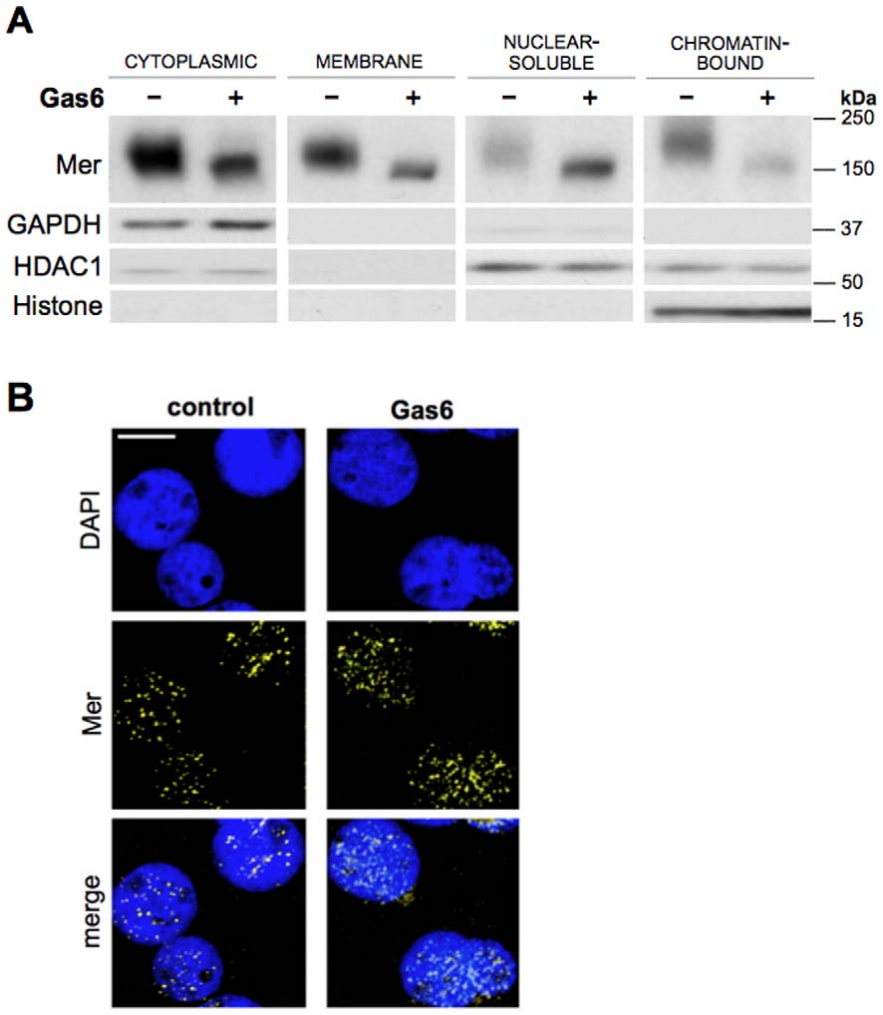
Prolonged Gas6 exposure alters Mer localization within the nuclear compartments. Jurkat cells were treated with 200 nM Gas6 (+) or vehicle control (−) for 24 hours. (**A**) Western blot detection of Mer following subcellular fractionation of cells. The following antibodies were used to assess compartment specificity: GAPDH (cytoplasmic), HDAC1 (both nuclear-soluble and chromatin-bound), and Histone H3 (chromatin-bound). (**B**) Following the 24-hour exposure, fixed and permeabilized cells were subjected to immunofluorescent staining and Mer expression (*yellow*) was analyzed by confocal microscopy. Nuclei were stained with DAPI (*blue*). *Scale bar*, 5 µm.

To determine if Gas6 influenced Mer elsewhere within the cell, subcellular fractions were analyzed by western blot after treating cells for 24 hours with 200 nM Gas6 or vehicle control. Surprisingly, Mer displayed a distinct expression pattern within the nuclear compartments—a subcellular localization not previously described for Mer. Relative to control, Mer expression was enhanced in the nuclear-soluble fraction but diminished in the chromatin-bound compartment of Gas6-treated samples (Figure 5A), a reciprocal pattern of expression suggesting that total levels of nuclear Mer were not markedly changed between control- and Gas6-treated cells. Consistent with this observation, immunofluorescent imaging substantiated the nuclear presence of Mer, which remained relatively similar in cells exposed to Gas6 or vehicle control (Figure 5B).

### Nuclear compartments display distinct preferences for Mer glycoforms

**Figure 6:**
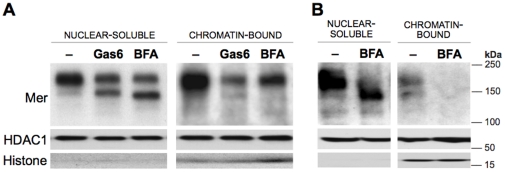
Nuclear compartments display preferential expression of different Mer glycoforms. Jurkat cells were cultured in the presence of 200 nM Gas6, 5 µg/ml BFA, or control (−) for the indicated times. Western blot detection of Mer in the nuclear-soluble and chromatin-bound fractions (**A**) collected after a 4-hour exposure, and (**B**) following a 24-hour treatment with BFA or control. Blots were also probed for HDAC1 and Histone H3 to ensure proper fractionation.

To investigate if altered expression of nuclear Mer directly resulted from Gas6 exposure or was primarily due to its partially glycosylated nature, western blot was used to evaluate Mer expression in the nuclear compartments of cells exposed to Gas6 or BFA. Cells were fractionated after treatment for 4 hours, a timepoint that would allow us to examine localization of the Gas6-responsive partial glycoform while fully glycosylated Mer remained the predominant form. If reduced expression of Mer in the chromatin-bound compartment were a Gas6-specific effect, we would expect to see decreased levels of both the full and partial glycoforms in the Gas6-treated samples but not the BFA-treated samples. If this effect were primarily due to altered Mer glycosylation, then expression of the partial glycoform would be largely excluded from the chromatin-bound compartment in both Gas6- and BFA-treated samples. Within the nuclear-soluble compartment, control cells expressed the partial Mer glycoform in minor proportion to the full glycoform; but in Gas6-treated cells, the partial glycoform existed in closer proportion to the full glycoform (Figure 6A). Similarly, BFA also resulted in nearly equal levels of partial and full Mer glycoforms in the nuclear-soluble fraction. In the chromatin-bound compartment, however, fully glycosylated Mer predominated in all samples, and the partial Mer glycoform expressed by BFA-treated cells was not detectable in this fraction. This effect was further pronounced in cells exposed to BFA for 24 hours (Figure 6B): by this point, the preexisting supply of full Mer glycoform had been replaced by a majority of newly translated protein bearing the BFA-restricted glycosylation, which was expressed in the nuclear-soluble fraction but noticeably absent in the chromatin-bound compartment.

### Mer contains conserved nuclear localization and export signals

**Figure 7:**
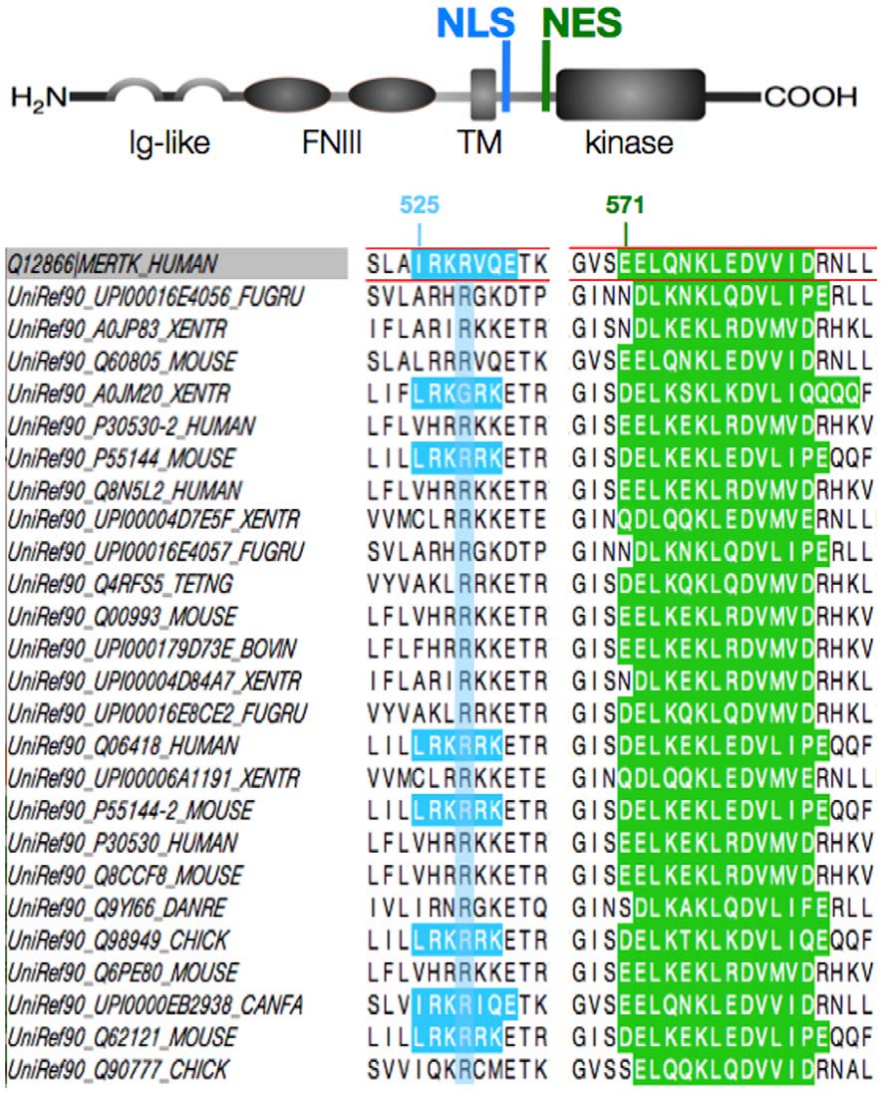
Mer contains conserved nuclear localization and export signals. Above, a diagram of Mer protein structure depicts the nuclear localization signal (NLS) and nuclear export signal (NES) within the juxtamembrane region of its intracellular domain. Additional functional domains are labeled accordingly (Ig, immunoglobulin; FNIII, fibronectin type III; TM, transmembrane). Below, sequence alignment highlights conservation of the C-extended monopartite variant NLS (*blue*) and the leucine-rich NES (*green*) among TAM receptors in both human and other species. Residue positions are indicated above the human Mer sequence outlined in red. Motif analysis and sequence alignment were performed using ELM [22] and Jalview software [49].

In additional support of a role for nuclear Mer, sequence motif and alignment analyses revealed that Mer contains putative nuclear localization and export signals (NLS and NES, respectively) within the juxtamembrane region of its intracellular domain, both of which are conserved among the TAM family members in humans and other species (Figure 7). The single cluster of three basic amino acid residues (RKR) at positions 526–528 in the Mer protein is classified as a conventional monopartite NLS; this motif, which was first identified in the SV40 large T antigen sequence [30], is often recognized and bound by importin (karyopherin) proteins that facilitate transport into the nucleus [22]. The nearby NES, residing at positions 572–583 in Mer, is predicted to bind CRM1 (also known as exportin1/Xpo1), a common export carrier protein [31], for transport out of the nucleus.

## Discussion

As previous experiments have focused on short-term characterization of Mer activation and functional effects associated with Mer expression, this paper for the first time examines the direct consequences of long-term Gas6 exposure on Mer. We used an *in vitro* model of prolonged Gas6 exposure to study Mer under conditions that more closely resemble the Gas6-replete environment that exists in the bone marrow [17]–[19] and plasma [12]–[16]—one in which the constant interaction between Gas6 and Mer presumably sustains downstream signaling activity and promotes leukemic cell survival *in vivo* [8], [19]. These studies provide insight into several novel aspects of Mer that redefine its function beyond the role of a signal transducer.

In human leukemia cell lines, continuous Gas6 exposure promoted expression of a partially N-glycosylated form of Mer, a glycoform that developed from *de novo* partial glycosylation of a newly synthesized protein. Collectively, the data from the glycan profiling and mechanistic studies suggest that persistent ligand exposure induces a switch to preferentially express the same partial Mer glycoform that normally exists as a minor form in the absence of Gas6. This idea is supported by the similarities in molecular weight and EndoH susceptibility (Figure 1C), which suggest a shared glycan profile; additionally, the comparable response of the partial glycoforms to tunicamycin (Figure 2C) indicates that they both utilize the same mechanism of post-translational modification, lending further support to this idea from a mechanistic perspective. The data obtained from tunicamycin-mediated glycosylation inhibition also provide novel insight into the receptor dynamics of Mer: by distinguishing newly synthesized (and non-glycosylated) proteins from mature receptors, we demonstrate that translation of new Mer occurs within a few hours. Based on the minimal amount of fully glycosylated Mer remaining after 24 hours of tunicamycin exposure, it is also evident that the majority of pre-existing receptor is recycled within this time frame. Although Mer dynamics still remain largely uncharacterized, this finding has important implications for drug design, as inhibitors with shorter half-lives would not likely have a sustained effect on Mer activity.

While Gas6-favored expression of the partial glycoform was associated with diminished levels of Mer on the cell surface (Figure 3C), exposure to BFA or TM, which restrict or completely inhibit glycosylation, respectively, demonstrated that the reduction in surface Mer was likely due to its partially glycosylated state rather than a direct consequence of ligand exposure (Figure 4). We also now report expression of Mer in the nucleus, illustrated both by immunofluorescent imaging and subcellular fractionation experiments (Figures 5 and 6). That Mer is expressed in any of the nuclear compartments—and as an intact, rather than cleaved, receptor—is a novel finding for this protein, as well as one that has not yet been reported for either of the other TAM receptors. However, several other studies have established the presence of full-length RTKs in the nucleus [32], including members of the epidermal growth factor receptor (EGFR) family of proteins [33], fibroblast growth factor receptor [34]–[36], insulin receptor [37], and insulin-like growth factor-1 receptor [38]. Expression of c-Met [39] and RON [40], receptors closely related to the TAM family [41], has also been demonstrated in the nucleus.

Although the conserved nature of the NLS and NES (Figure 7) suggest that Axl and Tyro3 may also localize to the nucleus, our efforts presently aim to determine the mechanistic bases of Mer translocation to the nucleus; as nuclear RTKs are associated with various changes in biologic activity [42], we are also focused on elucidating the functional significance of nuclear Mer expression. Based on reports of nuclear RTK-related effects on gene expression—including regulation by direct transactivation as well as through complexed interactions [40], [43]–[45]—ChIP-Seq profiling experiments are currently underway to identify regions of DNA influenced by Mer, which is present within the chromatin-bound fraction (Figures 5 and 6).

Several studies have reported differential glycosylation of Mer [5], [21], [23] but none have explored the functional effects associated with this modification. The contrasting patterns of glycoform expression observed within each nuclear fraction (Figure 6) suggest that the nuclear compartments may exhibit distinct preferences for specific Mer glycoforms. This glycoform-influenced nuclear sublocalization indicates functional consequences of Mer glycosylation, suggesting that particular N-glycan modifications on Mer may influence its affinity for DNA and/or partner proteins within the nucleus. Our data also highlight that Mer does not exclusively occupy the subcellular locales predicted by its glycosylation profile, suggesting that such modifications do not always restrict or define function. This idea is supported by a previous study emphasizing how the functional maturity of a protein is not necessarily defined by its degree of glycosylation: Krysov et al. found that despite the mannosylated, so-called “immature” nature of the IgM μ chain expressed in chronic lymphocytic leukemia samples, increased expression of this partial glycoform—which could be induced by persistent antigen exposure—retained the same functional capabilities as its fully glycosylated, “mature” counterpart [46].

Our observations widen the scope of potential Mer function and supplement its once-singular role as a surface receptor. Both the presence of Mer in the nucleus and the kinase-independent formation of the partial glycoform suggest that the oncogenic effects of Mer may, at least in part, be mediated through mechanisms involving its nuclear localization and not exclusively through increased activation at the cell surface. While previous studies have established Mer as an attractive therapeutic target in leukemia, the current focus on developing Mer-specific inhibitors emphasizes the importance of characterizing Mer function as thoroughly as possible, as the ability to effectively target Mer is limited by our understanding of its functions. The novel finding of Mer expression in the nucleus—and especially the presence of nuclear localization and export sequences—broadens the realm of potential approaches to target Mer for therapeutic purposes.

## Materials and Methods

### Cell culture and treatments

The Jurkat, HeLa, and HEK 293 cell lines were obtained from the American Type Culture Collection (ATCC), and HPB-ALL, 697, and NB4 cell lines from the German Collection of Microorganisms and Cell Cultures (DSMZ). Cells were maintained in RPMI 1640 medium (VWR) supplemented with 10% fetal bovine serum (FBS), 100 IU/L penicillin and 10 µg/ml streptomycin at 37°C in a humidified atmosphere containing 5% CO_2_. For experiments, cells were plated at an initial density of 1.0–1.3×10^6^ cells/ml in multi-well polystyrene tissue culture plates and incubated with equal volumes of treatments prepared as 10× stock solutions in serum-free RPMI. Recombinant human Gas6 (885-GS, R&D Systems) was reconstituted to 2 µM (139 µg/ml) immediately before adding to cells. Since Gas6 was lyophilized by the manufacturer from a solution containing Tris, NaCl, and Citrate, these additional components were included in preparation of vehicle control, which contained the same concentrations present in the volume of reconstituted Gas6. Tunicamycin (TM) and Brefeldin A (BFA) were purchased from Sigma.

### Preparation of lysates and cell fractions

After collecting cells by centrifugation, whole-cell lysates were prepared on ice using lysis buffer (50 mM HEPES pH 7.5, 150 mM NaCl, 10 mM EDTA, 10% glycerol, and 1% Triton X-100) supplemented with Halt Protease and Phosphatase Inhibitor Cocktail (Pierce). Cell fractions were isolated by stepwise lysis of cells using the Subcellular Protein Fractionation Kit (Pierce) according to manufacturer protocol. Protein concentrations were determined by 660 nm Protein Assay (Pierce).

### Glycosidase treatment

Lysates were denatured for 10 minutes at 100°C with Glycoprotein Denaturing Buffer and then digested with either peptide:N-glycosidase F (PNGaseF) or endoglycosidase H (EndoH) for 2 hours at 37°C in the appropriate buffer according to manufacturer protocol (New England BioLabs). Lysate aliquots incubated under the same conditions in the absence of enzymes served as controls. Samples were subsequently prepared for western blotting as described below.

### Western blotting

Normalized amounts of total protein were denatured by boiling in sample buffer (62 mM Tris pH 6.8, 0.7 M 2-mercaptoethanol, 10% glycerol, 2% SDS, and trace amount of bromophenol blue) prior to resolving by Tris-Glycine SDS-PAGE (Invitrogen). Proteins were transferred to PVDF membranes (Perkin-Elmer) and probed with the indicated antibodies using the rapid immunodetection method described previously [47]. Blots were washed with TBST (Tris-buffered saline containing 0.05% Tween-20) after primary and secondary immunoblotting steps. Proteins were detected by enhanced chemiluminescence (Perkin-Elmer) and imaged on film. All antibodies were diluted according to manufacturer recommendations in TBST containing 1% (w/v) nonfat milk. The anti-Mer antibody (#1633-1, Epitomics) was raised against a synthetic peptide mapping between amino acids 20 and 50 of human MerTK (Swiss Prot Q12866), a region within the extracellular domain that does not contain any putative N-glycosylation sites, and control experiments demonstrating specificity of this antibody for Mer in immunoblotting analyses are shown in Figure S3A. Antibodies used to detect Tubulin (#2125), phospho-p44/42 MAPK (phospho-Erk1/2, Thr202/Tyr204, #9106), p44/42 MAPK (Erk1/2, #9102), HDAC1 (#5356), GAPDH (#2118), Histone H3 (#4499), and Tyro3 (#5585) were purchased from Cell Signaling Technology; the anti-Axl antibody (AF154) was purchased from R&D Systems and the HRP-conjugated anti-actin antibody (sc-1616) from Santa Cruz Biotechnology. Primary antibodies were labeled with HRP-conjugated secondary antibodies (donkey anti-rabbit, 711-035-152; goat anti-mouse, 115-035-062) purchased from Jackson ImmunoResearch.

### Flow cytometric analysis of surface Mer expression

10^6^ cells were collected by centrifugation and kept at 4°C for the duration of the protocol. Phosphate-buffered saline (PBS) containing 1% FBS and 0.09% sodium azide was used for all wash and staining steps. After two washes, cells were incubated for 30 minutes with a 1∶50 dilution of Mer590, a monoclonal antibody directed against the Mer extracellular domain produced in a murine hybridoma as previously described [48], and washed twice more before staining with a 1∶50 dilution of phycoerythrin (PE)-labeled anti-mouse IgG (115-116-146, Jackson ImmunoResearch) for 30 minutes. Cells were washed four times and then resuspended in cold PBS. Fluorescence was measured using a FC500 flow cytometer with CXP data analysis software (Beckman Coulter). Control experiments demonstrating specificity of this antibody for Mer are shown in Figure S3C.

### Immunofluorescence microscopy

Cells were collected by centrifugation and kept on ice during all staining and wash steps. After washing twice in PBS containing 1% FBS, cells were fixed and permeabilized for 20 minutes with Cytofix/Cytoperm Solution and washed twice more with Perm/Wash Buffer (BD Biosciences). Perm/Wash Buffer was used for all subsequent staining and wash steps. Permeabilized cells were stained for intracellular Mer using a 1∶75 dilution of anti-Mer antibody (#1633-1, Epitomics) for 45 minutes, washed twice, and incubated in a 1∶200 dilution of DyLight549-labeled anti-rabbit secondary antibody (Jackson ImmunoResearch) for 25 minutes. After four washes, cells were resuspended in cold PBS, adhered to poly-l-lysine–coated coverslips (BD Biosciences) for 10 minutes at room temperature, and then affixed to glass slides with a drop of mounting reagent (Prolong Antifade Gold with DAPI, Invitrogen). Stained cells were visualized on a Zeiss LSM 510 META confocal microscope and all images were processed using LSM image browser software (Zeiss). Control experiments demonstrating specificity of this antibody for Mer in immunofluorescence imaging analyses are shown in Figure S3B.

### Plasmid construction and transfection

Mer add-back DNA constructs were produced using standard PCR methods. The coding region of Mer cDNA (Open Biosystems) was amplified with *HindIII* and *NotI* restriction sites and cloned into the pLNCX2 plasmid (Clontech). The kinase-dead Mer mutant, K619R (MerKD), was produced using a QuickChange site-directed mutagenesis kit (Stratagene) with an analogous DNA construct in pcDNA3 (Invitrogen), then subcloned into pLNCX2. Constructs were sequenced to verify the mutation and presence of the open reading frame.

Stable Jurkat add-back cell lines were developed using the LRCX retroviral gene expression system (Clontech) and procedures outlined in the Retrovirus Gene Transfer and Expression User Manual. Briefly, GP2-293 packaging cells (Clontech) were transfected with pVSV-G and either MerWT-pLNCX2, MerKD-pLNCX2 or pLNCX2. Virus was collected at 48 and 72 hours post-transfection, filtered and concentrated, and added to Mer-knockdown Jurkat cells (constructed to stably express shRNA vectors in WT Jurkat cells using the same methods and shRNA constructs (Open Biosystems) as described previously for the 697 and REH human B-ALL cell lines [10]) in the presence of polybrene (8 µg/ml ) for 18 hours. Selection began at 48 hours post-transduction with 400 µg/ml G418.

### Statistical analysis

Unless otherwise noted, all data are representative of three independent experiments. Statistical significance of results, defined by a *P*-value<0.05, was determined using Prism (Version 5; GraphPad Software). Details regarding specific analyses are indicated in the figure legends. Sequence motif analyses and alignments were performed using ELM (eukaryotic linear motif) resource [22] and Jalview software [49].

## Supporting Information

Figure S1 **Processes underlying Gas6-favored expression of the partial Mer glycoform.** Human leukemia cell lines were cultured for the indicated times in the presence of 200 nM Gas6 (+) or control (−), and Mer expression was detected by western blot of whole-cell lysates. Blots were probed for Tubulin to assess loading. (**A**) HPB-ALL cells were exposed to Gas6 for 24 hours in media containing either 10% FBS or 0% FBS. (**B**) 697 cells were exposed to a single dose of Gas6 and collected after 48, 72, or 96 hours. Similar results were also observed for Jurkat cells (not shown).
(TIF)

Figure S2 **Gas6-induced expression of the partial Mer glycoform does not require kinase activity.** Jurkat cells stably expressing a Mer add-back construct containing either wild type (WT) or a kinase-dead (K619R) form of Mer were exposed to 200 nM Gas6 (+) or vehicle control (−) for the indicated times and collected for western blot analysis of protein expression. (**A**) Validation of kinase-inactivating mutation: Erk1/2 phosphorylation (p-Erk1/2), an indicator of Mer activation, is enhanced in cells expressing WT, but not kinase-dead, Mer following a 10-minute Gas6 stimulation. (**B**) Similar to the effects observed with WT Mer, cells expressing kinase-dead Mer preferentially express the partial Mer glycoform after an 18-hour exposure to 200 nM Gas6.
(TIF)

Figure S3 **Both anti-Mer antibodies specifically recognize the Mer receptor tyrosine kinase.** All collection and detection methods were performed as described in the “Materials and Methods” section. (**A**) TAM receptor expression was detected by western blot of whole-cell lysates collected from three human cell lines—HeLa (cervical carcinoma), Jurkat (T-ALL), and NB4 (AML FAB M3). Each cell line displays a distinct pattern of TAM receptor expression, indicated above in the western blot and below in a summary table describing the presence (+) or absence (−) of each TAM receptor (grey shading represents lower expression levels relative to other cell lines). These data demonstrate that the anti-MerTK antibody (#1633-1, Epitomics) is specific for Mer and does not cross-react with either of the other TAM receptors. (**B**) The rabbit monoclonal anti-MerTK antibody (#1633-1, Epitomics) displays similar specificity in immunofluorescence staining. *Left:* Mer expression was determined by confocal imaging of Jurkat cells stably expressing shRNA directed against GFP (shControl, non-targeting control) or Mer (shMer1A) following immunofluorescence staining for Mer, and WT Jurkat cells stained only with secondary antibody (DyLight549) serve to demonstrate lack of non-specific binding. Merged images of Mer (*yellow*) and DAPI (*blue*) are shown and are representative of four independent experiments. *Right:* Immunoblot detection of Mer in WT, shControl, and shMer1A Jurkat cells (using the same anti-MerTK antibody) serves as a reference for confocal images and demonstrates sufficient shRNA-mediated knockdown of Mer. (**C**) Surface expression of Mer was assessed by flow cytometry after staining with the mouse monoclonal Mer590 antibody and subsequent incubation with a PE-conjugated anti-mouse secondary antibody. *Left:* Surface Mer was measured in Jurkat cells stably expressing either the shControl (*red*) or shMer1A (*blue*) construct, and another set of shControl cells were incubated with isotype-matched mouse IgG_1_ (*grey*) and then stained with secondary antibody as a negative control. The decreased level of Mer signal in shMer1A cells relative to shControl cells reflects a loss of antibody labeling due to Mer-knockdown and demonstrates specificity of the Mer590 antibody. *Middle:* A similar loss of Mer590 antibody labeling is seen in HEK 293 cells—which also express Tyro3, as well as Axl to a lesser extent, in addition to Mer (western blot, *right*)—following stable shRNA-mediated Mer knockdown relative to WT cells (immunoblot detection of Mer in each cell line shown above flow cytometry histogram). Thus, the loss of Mer590 labeling in Mer-knockdown HEK 293 cells, despite the presence of other TAM receptors, further demonstrates Mer590 antibody specificity for Mer.
(TIF)

## References

[1] Linger RMA, Keating AK, Earp HS, Graham DK (2008). TAM receptor tyrosine kinases: biologic functions, signaling, and potential therapeutic targeting in human cancer.. Adv Cancer Res.

[2] Chen J, Carey K, Godowski PJ (1997). Identification of Gas6 as a ligand for Mer, a neural cell adhesion molecule related receptor tyrosine kinase implicated in cellular transformation.. Oncogene.

[3] Linger RMA, Keating AK, Earp HS, Graham DK (2010). Taking aim at Mer and Axl receptor tyrosine kinases as novel therapeutic targets in solid tumors.. Expert Opin Ther Targets.

[4] Brandão L, Migdall-Wilson J, Eisenman K, Graham DK (2011). TAM Receptors in Leukemia: Expression, Signaling, and Therapeutic Implications.. Crit Rev Oncog.

[5] Graham DK, Salzberg DB, Kurtzberg J, Sather S, Matsushima GK (2006). Ectopic expression of the proto-oncogene Mer in pediatric T-cell acute lymphoblastic leukemia.. Clin Cancer Res.

[6] Yeoh EJ, Ross ME, Shurtleff SA, Williams WK, Patel D (2002). Classification, subtype discovery, and prediction of outcome in pediatric acute lymphoblastic leukemia by gene expression profiling.. Cancer Cell.

[7] Graham DK, Dawson TL, Mullaney DL, Snodgrass HR, Earp HS (1994). Cloning and mRNA expression analysis of a novel human protooncogene, c-mer.. Cell Growth Differ.

[8] Keating AK, Salzberg DB, Sather S, Liang X, Nickoloff S (2006). Lymphoblastic leukemia/lymphoma in mice overexpressing the Mer (MerTK) receptor tyrosine kinase.. Oncogene.

[9] Keating AK, Kim GK, Jones AE, Donson AM, Ware K (2010). Inhibition of Mer and Axl receptor tyrosine kinases in astrocytoma cells leads to increased apoptosis and improved chemosensitivity.. Molecular Cancer Therapeutics.

[10] Linger RMA, DeRyckere D, Brandão L, Sawczyn KK, Jacobsen KM (2009). Mer receptor tyrosine kinase is a novel therapeutic target in pediatric B-cell acute lymphoblastic leukemia.. Blood.

[11] Guttridge KL, Luft JC, Dawson TL, Kozlowska E, Mahajan NP (2002). Mer receptor tyrosine kinase signaling: prevention of apoptosis and alteration of cytoskeletal architecture without stimulation or proliferation.. J Biol Chem.

[12] Balogh I, Hafizi S, Stenhoff J, Hansson K, Dahlback B (2005). Analysis of Gas6 in human platelets and plasma.. Arteriosclerosis, thrombosis, and vascular biology.

[13] Borgel D, Clauser S, Bornstain C, Bièche I, Bissery A (2006). Elevated growth-arrest-specific protein 6 plasma levels in patients with severe sepsis.. Critical care medicine.

[14] Burnier L, Borgel D, Angelillo-Scherrer A, Fontana P (2006). Plasma levels of the growth arrest-specific gene 6 product (Gas6) and antiplatelet drug responsiveness in healthy subjects.. Journal of thrombosis and haemostasis : JTH.

[15] Clauser S, Bachelot-lozat C, Fontana P, Gaussem P, Remones V (2006). Physiological plasma Gas6 levels do not influence platelet aggregation.. Arteriosclerosis, thrombosis, and vascular biology.

[16] Hung YJ, Lee CH, Chu NF, Shieh YS (2010). Plasma protein growth arrest-specific 6 levels are associated with altered glucose tolerance, inflammation, and endothelial dysfunction.. Diabetes care.

[17] Avanzi GC, Gallicchio M, Cavalloni G, Gammaitoni L, Leone F (1997). GAS6, the ligand of Axl and Rse receptors, is expressed in hematopoietic tissue but lacks mitogenic activity.. Exp Hematol.

[18] Dormady SP, Zhang XM, Basch RS (2000). Hematopoietic progenitor cells grow on 3T3 fibroblast monolayers that overexpress growth arrest-specific gene-6 (GAS6).. Proc Natl Acad Sci USA.

[19] Shiozawa Y, Pedersen EA, Taichman RS (2010). GAS6/Mer axis regulates the homing and survival of the E2A/PBX1-positive B-cell precursor acute lymphoblastic leukemia in the bone marrow niche.. Exp Hematol.

[20] Sather S, Kenyon KD, Lefkowitz JB, Liang X, Varnum BC (2007). A soluble form of the Mer receptor tyrosine kinase inhibits macrophage clearance of apoptotic cells and platelet aggregation.. Blood.

[21] Graham DK, Bowman GW, Dawson TL, Stanford WL, Earp HS (1995). Cloning and developmental expression analysis of the murine c-mer tyrosine kinase.. Oncogene.

[22] Gould CM, Diella F, Via A, Puntervoll P, Gemund C (2010). ELM: the status of the 2010 eukaryotic linear motif resource.. Nucleic Acids Res.

[23] Feng W, Yasumura D, Matthes MT, LaVail MM, Vollrath D (2002). Mertk triggers uptake of photoreceptor outer segments during phagocytosis by cultured retinal pigment epithelial cells.. J Biol Chem.

[24] Maley F, Trimble RB, Tarentino AL, Plummer THJ (1989). Characterization of glycoproteins and their associated oligosaccharides through the use of endoglycosidases.. Analytical biochemistry.

[25] Roth J (2002). Protein N-glycosylation along the secretory pathway: relationship to organelle topography and function, protein quality control, and cell interactions.. Chemical reviews.

[26] Anwar A, Keating AK, Joung D, Sather S, Kim GK (2009). Mer tyrosine kinase (MerTK) promotes macrophage survival following exposure to oxidative stress.. J Leukoc Biol.

[27] Brown JR, Crawford BE, Esko JD (2007). Glycan antagonists and inhibitors: a fount for drug discovery.. Critical reviews in biochemistry and molecular biology.

[28] Wellen KE, Lu C, Mancuso A, Lemons JM, Ryczko M (2010). The hexosamine biosynthetic pathway couples growth factor-induced glutamine uptake to glucose metabolism.. Genes Dev.

[29] Klausner RD, Donaldson JG, Lippincott-Schwartz J (1992). Brefeldin A: insights into the control of membrane traffic and organelle structure.. J Cell Biol.

[30] Kalderon D, Roberts BL, Richardson WD, Smith AE (1984). A short amino acid sequence able to specify nuclear location.. Cell.

[31] Fornerod M, Ohno M, Yoshida M, Mattaj IW (1997). CRM1 is an export receptor for leucine-rich nuclear export signals.. Cell.

[32] Carpenter G, Liao HJ (2009). Trafficking of receptor tyrosine kinases to the nucleus.. Experimental cell research.

[33] Wang YN, Yamaguchi H, Hsu JM, Hung MC (2010). Nuclear trafficking of the epidermal growth factor receptor family membrane proteins.. Oncogene.

[34] Stachowiak MK, Moffett J, Maher P, Tucholski J, Stachowiak EK (1997). Growth factor regulation of cell growth and proliferation in the nervous system. A new intracrine nuclear mechanism.. Molecular neurobiology.

[35] Stachowiak MK, Fang X, Myers JM, Dunham SM, Berezney R (2003). Integrative nuclear FGFR1 signaling (INFS) as a part of a universal “feed-forward-and-gate” signaling module that controls cell growth and differentiation.. Journal of Cellular Biochemistry.

[36] Bryant DM, Stow JL (2005). Nuclear translocation of cell-surface receptors: lessons from fibroblast growth factor.. Traffic.

[37] Seol KC, Kim SJ (2003). Nuclear matrix association of insulin receptor and IRS-1 by insulin in osteoblast-like UMR-106 cells.. Biochem Biophys Res Commun.

[38] Sehat B, Tofigh A, Lin Y, Trocmé E, Liljedahl U (2010). SUMOylation mediates the nuclear translocation and signaling of the IGF-1 receptor.. Science signaling.

[39] Gomes DA, Rodrigues MA, Leite MF, Gomez MV, Varnai P (2008). c-Met must translocate to the nucleus to initiate calcium signals.. J Biol Chem.

[40] Liu HS, Hsu PY, Lai MD, Chang HY, Ho CL (2010). An unusual function of RON receptor tyrosine kinase as a transcriptional regulator in cooperation with EGFR in human cancer cells.. Carcinogenesis.

[41] Robinson DR, Wu YM, Lin SF (2000). The protein tyrosine kinase family of the human genome.. Oncogene.

[42] Lo HW, Hung MC (2006). Nuclear EGFR signalling network in cancers: linking EGFR pathway to cell cycle progression, nitric oxide pathway and patient survival.. Br J Cancer.

[43] Xie Y, Hung MC (1994). Nuclear localization of p185neu tyrosine kinase and its association with transcriptional transactivation.. Biochem Biophys Res Commun.

[44] Lin SY, Makino K, Xia W, Matin A, Wen Y (2001). Nuclear localization of EGF receptor and its potential new role as a transcription factor.. Nat Cell Biol.

[45] Wang SC, Lien HC, Xia W, Chen IF, Lo HW (2004). Binding at and transactivation of the COX-2 promoter by nuclear tyrosine kinase receptor ErbB-2.. Cancer Cell.

[46] Krysov S, Potter KN, Mockridge CI, Coelho V, Wheatley I (2010). Surface IgM of CLL cells displays unusual glycans indicative of engagement of antigen in vivo.. Blood.

[47] Mansfield MA (1995). Rapid immunodetection on polyvinylidene fluoride membrane blots without blocking.. Analytical biochemistry.

[48] Rogers AEJ, Le JP, Sather S, Pernu BM, Graham DK (2011). Mer receptor tyrosine kinase inhibition impedes glioblastoma multiforme migration and alters cellular morphology.. Oncogene.

[49] Waterhouse AM, Procter JB, Martin DM, Clamp M, Barton GJ (2009). Jalview Version 2–a multiple sequence alignment editor and analysis workbench.. Bioinformatics.

