# Supplementary figures and images for: Prolonged Exposure to a Mer Ligand in Leukemia: Gas6 Favors Expression of a Partial Mer Glycoform and Reveals a Novel Role for Mer in the Nucleus
Source: PLoS One. 2012 Feb 20;7(2):e31635. doi: 10.1371/journal.pone.0031635 (PMC3282750; doi:10.1371/journal.pone.0031635)

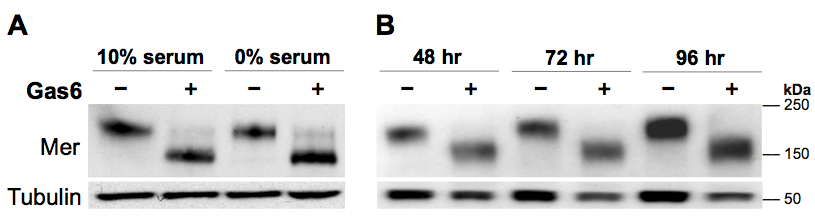

Supplement: Figure S1 — Processes underlying Gas6-favored expression of the partial Mer glycoform. Human leukemia cell lines were cultured for the indicated times in the presence of 200 nM Gas6 (+) or control (−), and Mer expression was detected by western blot of whole-cell lysates. Blots were probed for Tubulin to assess loading. (A) HPB-ALL cells were exposed to Gas6 for 24 hours in media containing either 10% FBS or 0% FBS. (B) 697 cells were exposed to a single dose of Gas6 and collected after 48, 72, or 96 hours. Similar results were also observed for Jurkat cells (not shown). (TIF) [file pone.0031635.s001.tif]

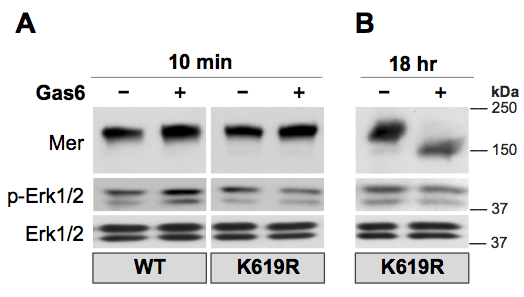

Supplement: Figure S2 — Gas6-induced expression of the partial Mer glycoform does not require kinase activity. Jurkat cells stably expressing a Mer add-back construct containing either wild type (WT) or a kinase-dead (K619R) form of Mer were exposed to 200 nM Gas6 (+) or vehicle control (−) for the indicated times and collected for western blot analysis of protein expression. (A) Validation of kinase-inactivating mutation: Erk1/2 phosphorylation (p-Erk1/2), an indicator of Mer activation, is enhanced in cells expressing WT, but not kinase-dead, Mer following a 10-minute Gas6 stimulation. (B) Similar to the effects observed with WT Mer, cells expressing kinase-dead Mer preferentially express the partial Mer glycoform after an 18-hour exposure to 200 nM Gas6. (TIF) [file pone.0031635.s002.tif]

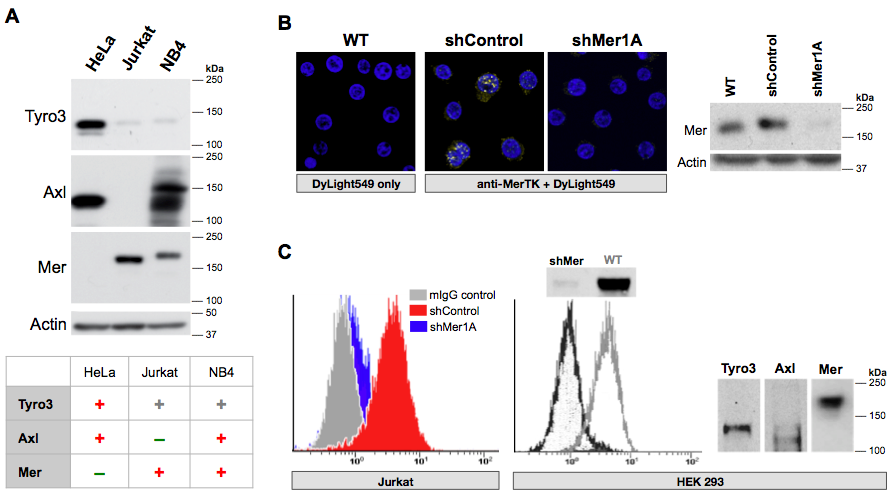

Supplement: Figure S3 — Both anti-Mer antibodies specifically recognize the Mer receptor tyrosine kinase. All collection and detection methods were performed as described in the “Materials and Methods” section. (A) TAM receptor expression was detected by western blot of whole-cell lysates collected from three human cell lines—HeLa (cervical carcinoma), Jurkat (T-ALL), and NB4 (AML FAB M3). Each cell line displays a distinct pattern of TAM receptor expression, indicated above in the western blot and below in a summary table describing the presence (+) or absence (−) of each TAM receptor (grey shading represents lower expression levels relative to other cell lines). These data demonstrate that the anti-MerTK antibody (#1633-1, Epitomics) is specific for Mer and does not cross-react with either of the other TAM receptors. (B) The rabbit monoclonal anti-MerTK antibody (#1633-1, Epitomics) displays similar specificity in immunofluorescence staining. Left: Mer expression was determined by confocal imaging of Jurkat cells stably expressing shRNA directed against GFP (shControl, non-targeting control) or Mer (shMer1A) following immunofluorescence staining for Mer, and WT Jurkat cells stained only with secondary antibody (DyLight549) serve to demonstrate lack of non-specific binding. Merged images of Mer (yellow) and DAPI (blue) are shown and are representative of four independent experiments. Right: Immunoblot detection of Mer in WT, shControl, and shMer1A Jurkat cells (using the same anti-MerTK antibody) serves as a reference for confocal images and demonstrates sufficient shRNA-mediated knockdown of Mer. (C) Surface expression of Mer was assessed by flow cytometry after staining with the mouse monoclonal Mer590 antibody and subsequent incubation with a PE-conjugated anti-mouse secondary antibody. Left: Surface Mer was measured in Jurkat cells stably expressing either the shControl (red) or shMer1A (blue) construct, and another set of shControl cells were incubated with isotype-matched mouse IgG1 [file pone.0031635.s003.tif]
